# The promise of microRNA-based therapies in Alzheimer’s disease: challenges and perspectives

**DOI:** 10.1186/s13024-021-00496-7

**Published:** 2021-11-06

**Authors:** Hannah Walgrave, Lujia Zhou, Bart De Strooper, Evgenia Salta

**Affiliations:** 1 VIB Center for Brain & Disease Research, Leuven, KU, Leuven, Belgium; 2 Department of Neurosciences, Leuven Brain Institute, Leuven, Belgium; 3 Division of Janssen Pharmaceutica NV, Discovery Neuroscience, Janssen Research and Development, Beerse, Belgium; 4 UK Dementia Research Institute at University College London, London, UK; 5 Netherlands Institute for Neuroscience, Amsterdam, The Netherlands

**Keywords:** microRNA, Alzheimer’s disease, therapeutics, neurodegenerative diseases

## Abstract

Multi-pathway approaches for the treatment of complex polygenic disorders are emerging as alternatives to classical monotarget therapies and microRNAs are of particular interest in that regard. MicroRNA research has come a long way from their initial discovery to the cumulative appreciation of their regulatory potential in healthy and diseased brain. However, systematic interrogation of putative therapeutic or toxic effects of microRNAs in (models of) Alzheimer’s disease is currently missing and fundamental research findings are yet to be translated into clinical applications. Here, we review the literature to summarize the knowledge on microRNA regulation in Alzheimer’s pathophysiology and to critically discuss whether and to what extent these increasing insights can be exploited for the development of microRNA-based therapeutics in the clinic.

## Alzheimer’s disease complexity: on a quest for network-based approaches to therapy

Alzheimer’s disease (AD) was until recently perceived as a neuron-centric disorder with linearly evolving pathology, initiated by the deposition of β amyloid (Aβ) peptides followed by the accumulation of hyperphosphorylated TAU (pTAU) into neurofibrillary tangles and ultimately leading to a full-spectrum neurodegenerative condition with prominent dementia after a long period of 10-20 years [1–4]. Novel molecular and genetic insights have challenged the unidirectional linearity of the pathogenic cascade in AD and underscored the significance of intertwined complex cellular pathways, gene networks and feed-forward regulatory loops that may differentially impact distinct pathogenic endophenotypes and cellular phases of the disease [4–7]. Multi-omics data suggest that the genetic risk of AD functionally translates into molecular networks involved in neuroinflammation, synaptic, lysosomal and phagocytic dysfunction, vascular and metabolic alterations and white matter changes [7–11]. Mapping the mechanistic heterogeneity and multifactorial nature of AD is a key challenge, given the current scarcity of effective disease-modifying monotherapies [12, 13]. Just as treating multiple pathways and molecular networks in other diseases, such as cancer and human immunodeficiency virus-1 has improved outcome [13–15], a similar network medicine approach can be envisaged for AD [10, 13, 16, 17]. The rationale for advancing combination therapy in AD was clearly laid out recently during the Alzheimer’s Association Research Roundtable meeting [18]. Combination therapies that can exert multiple effects on disease biology (e.g. anti-amyloid and/or anti-TAU and/or anti-inflammatory agents) have been recently considered and are currently in the drug development pipeline as stand-alone or add-on treatments to those already in the clinic (cholinesterase inhibitors and NMDA receptor antagonists) for AD (for a systematic review of ongoing AD clinical trials, see [13, 19, 20]). These combination schemes can either involve the multi-modal, combinatorial administration of more than one therapeutic agents (e.g. ALZT-OPT1: anti-amyloid & anti-inflammatory treatment, NCT02547818; ANAVEX2-73: anti-amyloid & anti-TAU & anti-inflammatory, NCT03790709) or the use of multifunctional molecules (e.g. Rasagiline: neuroprotective & anti-amyloid, NCT02359552).

MicroRNA-targeted therapeutics are particularly suited for such multi-targeting purposes, as they can typically ‘hit’ multiple sensitive nodes of various molecular cascades deregulated in disease conditions. MicroRNAs (miRNAs) are small, ~22nt-long, non-protein coding RNAs that induce posttranscriptional gene silencing by binding their complementary messenger RNA (mRNA) targets and inhibiting translation and/or inducing mRNA degradation [21, 22]. Under physiological conditions, miRNA-dependent gene regulation acts to ensure precise protein output and minimal protein expression noise [23, 24]. miRNAs are also able to sense and rapidly respond to the presence of stressors in their microenvironment, providing molecular robustness to cellular stress and restoring tissue homeostasis [25–27]. While miRNAs may only modestly repress individual targets, their regulatory power relies on the context-specific, synergistic cross-talk between subsets of miRNAs over prioritized sets of transcripts [28–32]. Under pathological conditions, as it is the case in cancer tissue or in neurodegeneration, this network-based mode of gene regulation converges onto distinct molecular pathways, which potently drive disease phenotypes [30, 33, 34]. Yet, profiling the impact of miRNA-based multi-targeting therapeutic strategies in AD with respect to efficacy and toxicity remains a daunting task, which is reflected by the current sparsity of miRNA therapeutics undergoing clinical trials in AD, as we discuss below.

## Functional pleiotropy of microRNAs in the central nervous system: one molecule, several pathways

miRNAs in the central nervous system (CNS) control gene expression in various cell types and in a highly regulated time-, space-, and neuronal activity- dependent manner [35–39]. Emerging evidence points towards a complex multicellular gene silencing repertoire for several of the studied miRNAs in the brain (for a comprehensive review refer to [40]). The miRNA transport across different brain-resident cell types along with the periphery-CNS miRNA interchange further contribute to the intercellular signaling crosstalk [41, 42].

A prototype of the intricate multicellular miRNA functions in CNS are ‘NeurimmiRs’, a term collectively referring to miRNAs acting at the interface between the neuronal and the immune systems [43]. miR-124, miR-132 and miR-146 exemplify this sort of miRNA regulatory pleiotropy with direct implications in neurodegeneration [30, 43]. miR-124 is a brain-specific miRNA regulating neurogenesis [44, 45], synaptic plasticity [46] and behavior [47]. In addition, its immunomodulatory role is exerted via the targeting of the transcription factor CCAAT/enhancer-binding protein-α, which converts microglia from an activated and inflammatory state to a quiescent phenotype [48]. Microglia endogenously express miRNA-124 and can also receive neuronally-derived exosomal miR-124 [49]. miR-124-mediated paracrine signaling between SH-SY5Y neuroblastoma cells overexpressing an AD mutation and microglia in culture induces a shift of microglial phenotype from an initially proinflammatory to a more regenerative state, re-establishing homeostasis [50].

The brain-enriched miR-132 is a key regulator of neuronal morphogenesis [51–55], synaptic plasticity [56–59], neuronal survival [60, 61] and cognition [62–66]. We recently reported a novel role for miR-132 in restoring hippocampal neurogenesis in the adult AD mouse brain by regulating several stages of the neurogenic process, including proliferation, differentiation, maturation and providing neurotrophic and neuroprotective support [67]. Emerging evidence suggests additional roles for miR-132 in immunomodulation, although extensive *in vivo* documentation in CNS is currently missing. More specifically, miR-132 exerts significant anti-inflammatory effects in monocytes and macrophages *in vitro* [63, 68]. In addition, miR-132 overexpression in the U251 human astrocytic cell line targets interleukin-1 receptor-associated kinase, resulting in decreased secretion of pro-inflammatory cytokines IL-1β and IL-6 [69]. *In vivo*, miR-132 targets acetylcholinesterase, thereby increasing the levels of acetylcholine, a key suppressor of pro-inflammatory cytokines [70]. Moreover, astrocytic and microglial miR-132 levels increase in epileptic rat and human brain as a protective response, while miR-132 transfection in human primary astrocytes represses expression of pro-inflammatory and pro-epileptogenic genes [71]. Although these observations support a role of miR-132 in the fine-tuning of inflammation, evidence of direct regulation of the innate immune response by miR-132 in the brain is currently lacking. Attempts to profile the baseline miR-132 levels in microglia and astrocytes *in vivo* and their response to AD onset and progression can provide an initial basis for further mechanistic assessment.

miR-146 is abundantly expressed in microglia and to a lesser extent in neurons and astrocytes. miR-146 represses the nuclear factor kappa-B (NF-κB) signaling pathway in several cell lines *in vitro* by directly targeting IRAK1 and TRAF6 [72–75]. miR-146 knockout mice fail to induce effective microglia-mediated phagocytosis in response to lipopolysaccharide pro-inflammatory stimulation, suggesting that miR-146 is essential for the microglial response to inflammation [76]. In addition, miR-146 induces pro-neurogenic effects, promoting neuronal differentiation and neuronal lineage commitment of human neural stem cells *in vitro* [77], while miR-146 knockout in mouse hippocampus impairs radial glia-like cell differentiation and causes severe memory impairment [78]. Interestingly, miR-146 was also shown to exert inflammation-mediated synaptic alterations in a non-cell autonomous manner via the functional intercellular crosstalk between neurons and microglia. More specifically, inflammatory microglia in culture shuttle miR-146 in extracellular vesicles to neurons, where it induces synaptic loss through targeting of Nlg and Syt1 [79]. Whether the immunomodulatory effects of miR-146 also occur in the brain under physiological or pathological conditions and if they may additionally mediate its impact on memory formation has not been addressed yet.

Evidently, considering NeurimmiRs to tackle both neuronal and immune aspects of AD pathogenesis is an attractive hypothesis. However, most of the studies discussed here do not concomitantly explore miRNA effects in both immune and neuronal functions in the brain. Moreover, current evidence is primarily based on ‘one miRNA-one target’ experimental design, which precludes the unbiased systematic mapping of both disease- and tolerability-relevant effects. In addition, miRNA effect sizes are often relatively small (20-30%) when assaying single targets. However, the network-based regulatory potential of miRNAs can be addressed in unbiased multi-target or genome-wide studies, where the global impact on multiple targets acting within one pathway or endophenotype is considered as a whole [32, 80, 81]. This novel approach to studying miRNAs elucidates the functional significance of miRNAs as network regulators, however, has not yet been widely applied in CNS systems.

## MicroRNAs in key AD pathways: targeting endophenotype complexity

miRNA profiles in the brain of AD patients are altered compared to healthy controls, often in a stage- and/or region- specific manner [82–87]. How these alterations impact disease onset and progression and whether they act as cause or effect along the disease trajectory remains unclear. Nevertheless, specific early miRNA aberrations along AD progression in human brain, indicate that disruption of miRNA homeostasis may act as a (co-)driver of certain pathological cascades [84]. Indeed, miRNAs have been shown to be responsive to a wide range of neuropathological processes, including oxidative stress, neuroinflammation, protein aggregation, and alterations in neuronal connectivity and plasticity, suggesting that miRNA-regulated molecular pathways may be interfering with pathology early on in the progression of neurological disorders [54, 68, 69, 88–94].

Of note, evidence of genetic association between miRNAs and AD is scarce and most of the pertinent studies are underpowered. Only a handful of single nucleotide polymorphisms have been identified to date in precursor miRNA sequences in genome-wide association studies of AD patient cohorts, of which rs2291418 in the miR-1229 precursor is one of the best studied examples [95–97]. miR-1229 targets SORL1, an AD risk gene involved in amyloid precursor protein (APP) processing. Although potentially very interesting, this association has not yet been functionally validated. Nevertheless, strong correlative evidence links miRNAs to several key AD endophenotypes in human brain. One of the best studied miRNAs in this series is miR-132, which is among the most consistently downregulated miRNAs in AD [84, 86, 98]. We and others have previously shown that miR-132 levels are anti-correlated with deposition of both intraneuronal hyperphosphorylated TAU and extracellular amyloid aggregation in the prefrontal cortex of human AD brain [33, 84, 98]. Interestingly, miR-132 expression variation explains 6.7% of the observed variance in histopathological AD endophenotypes in the Religious Order Study and the Rush Memory Aging Project. This actually outperforms the 6.1% of variance explained in the same patient cohorts by Apolipoprotein E4 (APOE4), the largest risk factor for AD [98]. In addition, miR-132 is among the core set of variables that explain the contribution of an individual’s polygenic risk score to cognitive impairment in AD [99]. This evidence suggests a putative functional link between miR-132 and amyloidosis/TAU pathology/dementia in human AD brain and offers a direct proof-of-principle as to how one miRNA may concomitantly regulate multiple pathways associated with AD pathophysiology.

miR-203 provides another interesting example of miRNAs potentially playing multilayered roles in AD [32]. miR-203 was identified as a hub regulator of the neuronal/synaptic- and microglial/inflammatory modules in the Tau^P301S^ tauopathy mouse model. Similar modules were also mapped in other mouse models of frontotemporal dementia (FTD) and in human postmortem FTD brain. While a set of targets involved in apoptotic cell death were shown to be regulated by miR-203, miR-203 *per se* has not been previously reported among the deregulated miRNAs in neurodegeneration. Hence, even though the notion of network regulation is a pivotal one, the conditions under which miR-203 may actively and potently regulate neuronal death still remain to be addressed.

Recently, a meta-analysis study identified a signature of 10 commonly deregulated miRNAs with predicted or previously validated neuroimmune functions, including miR-9-5p, miR-21-5p, the miR-29 family, miR-132-3p, miR-124-3p, miR-146a-5p, miR-155-5p, and miR-223-3p, across several neurodegenerative disorders ranging from AD to multiple sclerosis and prionopathies [30]. These correlative observations strongly suggest that miRNAs may represent prime candidates for intervention as common downstream regulators of functionally diverse molecular pathways in AD. Yet, while some of these miRNAs have been previously studied and are discussed here, experimental testing of newly identified correlations in the appropriate model systems is critical for mechanistic assessment prior to any consideration in drug development pipelines.

One of the main arguments used in favor of a putatively central role for miRNAs in AD, is that many of the molecules with central roles in disease pathogenesis, such as APP [100–112], β-secretase (BACE1) [104, 111, 113–124], APOE [125–127] and TAU [33, 61, 64, 128–130], are direct or indirect miRNA targets. We have previously demonstrated that miR-132 can bimodally regulate both amyloidosis and Tau phosphorylation via regulating one single target, ITPKB, acting upstream of both cascades [33]. The functional convergence of miR-132 regulation on repressing Tau pathology has been demonstrated via multiple additional targets acting on Tau phosphorylation, splicing or even *Tau* mRNA itself [64, 84, 131, 132]. Along with a role in suppressing neuronal apoptosis via direct regulation of PTEN, FOXO3a and P300, the multi-pathway regulatory repertoire of miR-132 in AD is among the most extensively studied and validated [60].

Of note, functional evidence for beneficial roles of miRNAs in AD suggests that modifying their expression might counteract pathology. Intracerebral infusion of lentiviral constructs expressing miR-188-5p in 5xFAD mice restores dendritic spine density and memory deficits in contextual fear conditioning and T-maze tests [93], while restoring miR-132 levels through direct delivery of miR-132 synthetic mimics into the brain of different AD mouse models (APP/PS1, 3xTg AD and APP^NL-G-F^ mice), ameliorates Aβ_40-42_ levels, Tau phosphorylation, deficits in adult hippocampal neurogenesis and cognition [33, 64, 67]. Additionally, stereotactic injections of lentiviral constructs expressing miR-338-5p in the dentate gyrus of 5xFAD mice, decreased BACE1 protein levels, Aβ_42_ and neuroinflammation, and rescued spatial memory deficits in the Morris water maze test [133]. More recently, non-invasive, nose-to-brain delivery of miR-146 synthetic antisense oligonucleotides in APP/PS1 mice, was shown to ameliorate amyloid and Tau pathologies, neuroinflammation and memory deficiency [134].

**Fig. 1:**
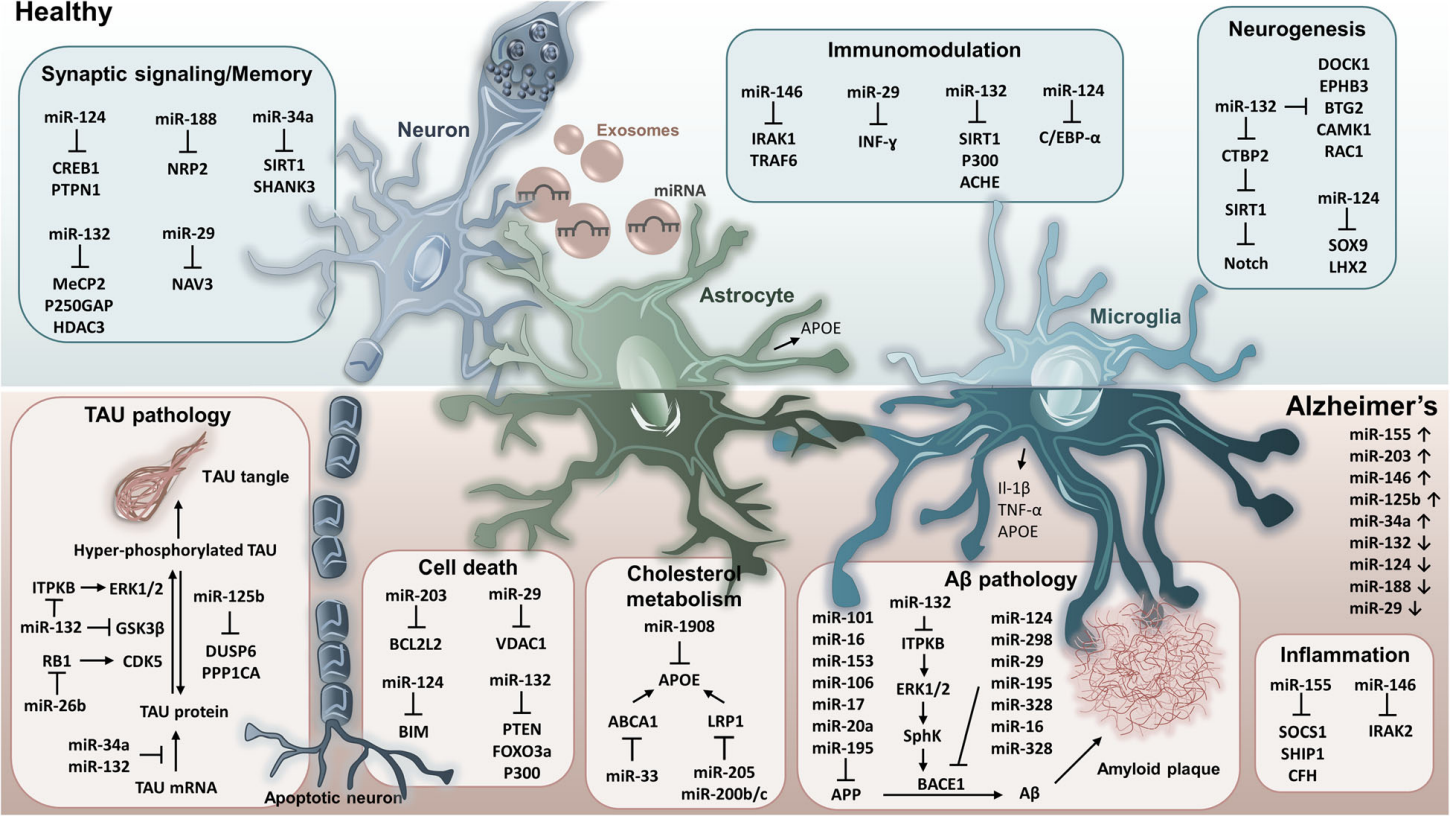
microRNAs regulate multiple AD-related cellular and molecular pathways. In healthy brain, miRNAs can maintain homeostasis through negative regulation of targets in neuronal and immune pathways. In Alzheimer’s brain, miRNAs are dysregulated impacting cellular and molecular cascades involved in AD endophenotypes.

Taken together, these observations provide proof-of-concept that modulating miRNA levels in AD brain may be considered therapeutically, as it could concomitantly modify multiple aspects of the pathology (Figure 1) and potentially lead to amelioration of memory deficits. Context-specificity is a significant aspect of miRNA biology, hence, results from studies using only *in vitro* systems or a single transgenic mouse line may not be easily extrapolated to describe widespread miRNA-mediated effects in AD. Along the same lines, increasing the levels of miRNA in a tissue or cell population, where it was previously absent or lowly expressed, may elicit ‘de novo’ target repression with unpredictable effects. In addition, in order to enable a therapeutic effect, careful dose titration to determine the required therapeutic window must be considered. We and others have shown that ‘supraphysiological’ (>3-fold) miR-132 levels following overexpression, may exert negative effects on memory [66, 67]. This narrow dose window is in line with the role of miRNAs as fine-tuners of their target’s protein levels around a physiological set-point, indicating that both too high and too low levels can be detrimental [94]. Yet, this aspect of manipulating miRNA levels in preclinical mouse models is often overlooked in the literature.

## MicroRNAs as biomarkers of Alzheimer’s disease

The remarkable stability of miRNAs in the extracellular environment and hence, in bodily fluids, together with the availability of sensitive methods for their detection and quantitation, has led to circulating miRNAs being widely employed as biomarkers for various human disorders [135]. Several miRNA-based diagnostic tests are already used in the clinic, mostly assessing panels of miRNAs, in different types of biological samples and conditions ranging from liver injury to cardiovascular disease and cancer [136–138].

In the AD field, currently available biomarkers include Aβ_40-42_, total TAU and phosphorylated TAU levels in the cerebrospinal fluid (CSF), along with brain imaging, such as positron emission tomography and structural magnetic resonance imaging scans to visualize Aβ/TAU deposits and atrophy, respectively [139–141]. More recently, also blood and plasma biomarkers have emerged as promising diagnostic tools, such as Aβ_40/42_ levels, plasma TAU phosphorylation at residue 181 (pTAU181) and plasma neurofilament light chain levels [142–148]. The use of peripheral miRNA levels as AD diagnostics is currently at relatively advanced stages of clinical development. miRNA profiles in CSF, blood, plasma and serum have been measured and compared between AD patients and healthy controls [83, 149–155]. Leidinger and colleagues have proposed a panel of 12 miRNAs in blood that discriminates AD from other CNS diseases and allows to distinguish between AD and MCI patients with an accuracy of 76%, which comes close to the 80% accuracy of blood pTau181 [144, 156]. The 12-miRNA signature could differentiate healthy from AD individuals with an accuracy of 93%, a specificity of 95% and a sensitivity of 92% [156]. These data were thereafter confirmed in independent patient cohorts and validated using machine learning approaches [157, 158], however, they have not been implemented in the clinical testing pipeline yet. Recently, a combination of 3 miRNAs and 3 Piwi-interacting RNAs (piRNA) isolated from CSF-circulating exosomes were found significantly altered in AD patients compared to controls. By adding the information of this miRNA/piRNA signature to CSF pTAU and Aβ_40/42_ ratio values, the classification accuracy for AD patients increased from 83% to 98% [159], suggesting that, possibly in combination with other existing biomarkers, the implementation of such signatures could contribute to stable, high-performance diagnostic tests for AD. Capitalizing on these observations, multiple products are currently in the pipeline by several companies [136]. CognimiR, a diagnostic panel of 24 brain-enriched and inflammation-associated miRNAs in blood, for early detection of mild cognitive impaired (MCI) and asymptomatic AD patients with 90% discrimination accuracy from controls (which compares with and outperforms the specificity levels achieved by plasma Aβ_40-42_ assessment [160]), was recently branded by DiamiR and is currently in phase I of clinical development [136]. Another miRNA-based diagnostic test for AD is being developed by Hummingbird Diagnostics, however, clinical validation has not been initiated yet [136]. Given that miRNAs in blood are easier to detect than those in CSF [161, 162], they have been found to change more consistently across independent cohorts of AD patients than CSF miRNAs [154], and there is a scarcity of standardized miRNA isolation protocols from CSF samples [161], it becomes obvious that the choice of biological fluid can be critical for the clinical development of miRNA diagnostics. Along with their minimal invasiveness, plasma- or serum-based assays may therefore be of advantage. In addition, elevated levels of miR-206 have been measured in the nasal mucosa of AD patients compared to healthy controls [163], while increased miR-200b-5p abundance was detected in tear fluid from AD patients [164]. Once validated in larger populations, these latter findings could represent novel promising non-invasive strategies for early diagnosis and further suggest that miRNAs could be harnessed for biomarker-guided drug development in AD. Future high-powered cohort studies implementing diverse patient cohorts will be required to further confirm these observations.

## Bringing microRNA therapeutics into the clinical practice: hurdles and challenges

Federal Drug Agency (FDA)-approved drugs for AD include cholinesterase inhibitors, memantine and just very recently, also the first disease-modifying drug, the monoclonal antibody aducanumab, which has been shown to lower Aβ levels [20, 165, 166]. Along with a long list of other agents currently undergoing clinical trials, these approaches all exhibit distinct differences to miRNA-based strategies (Table 1). The current gap in miRNA-related clinical studies as compared to other therapeutic strategies in AD, is indicative of a set of distinct limitations, which the development of miRNA-based therapeutics is currently facing.

**Table 1:** Examples of different types of therapeutic agents in clinical trials for Alzheimer's disease, each with their own advantages and disadvantages. For a complete list, see Cummings et al. (2021) [19]

Agent type	Name	Target type	Mechanism of action	Clinical stage	Advantages	Disadvantages
**Small molecule**	Donepezil Galantamine Rivastigmine	Cholinergic system	Cholinesterase inhibitor, increases level of neurotransmitter acetylcholine	FDA-approved	Often easy dosing, such as oral administration Can target extracellular and intracellular targets Some can cross the BBB Combination therapy possible/ongoing Faster clearance than mAbs, good to avoid some side-effects Stable	'One-drug, one target' Specificity can be dependent on binding-site, affinity, etc. Slow and laborious optimization
	Memantine	Glutamatergic system	N-methyl-D-aspartate (NMDA) receptor antagonist, affects glutamatergic transmission			
	LMTX (TRx0237)	TAU-targeting agent	TAU aggregation inhibitor	Phase III ongoing		
	ALZT-OP1	Inflammation-modifying agent	Combination therapy: ibuprofen is a nonsteroidal anti-inflammatory; cromolyn is a mast cell stabilizer with anti-Aβ effects	Phase III ongoing		
	Mastinib		Tyrosine kinase inhibitor, modulates neuroinflammation	Phase III completed		
**Immunotherapy/antibody/protein**	Aducanumab	Aβ-targeting agent	Monoclonal antibody, binds aggregated Aβ fibrils and soluble oligomers	FDA-approved	Targeted, specific therapeutics Available knowledge, plenty of antibody-based drugs approved Not a lot of toxicity due to humanization of antibody-based drugs	Only one monoclonal antibody has shown sufficient efficacy in humans so far (due to low effective dose in brain) Delivery issues, do not cross BBB Mostly extracellular targets, unstable, difficult manufacturing/slow and laborious optimization Need relative invasive intravenous or subcutaneous injections
	BAN2401		Monoclonal antibody, binds soluble Aβ protofibrils	Phase III ongoing		
	Gantenerumab		Monoclonal antibody, binds aggregated Aβ	Phase III ongoing		
	Gosuranemab	TAU-targeting agent	Monoclonal anti-TAU antibody, binds extracellular, N-terminal fragments of tau	Phase II ongoing		
**RNA-based**	AAVrh.10-APOE2	APOE	Viral delivery of APOE2	Phase I ongoing	Single administration, targeted delivery	Delivery issues, do not cross BBB Need invasive intathecal injections Cytotoxicity
	IONIS-MAPTRx	TAU-targeting agent	ASO, binds *TAU* mRNA and inhibits translation	Phase I/II ongoing	Easy to manufacture, targeted/specific therapeutic, can target at any site	
	miRNAs	Multi-targeting agent	miRNA mimic oligonucleotides (miRNA supplementation), miRNA antisense oligonucleotides (miRNA knockdown)	Preclinical	Simultaneous targeting of multiple AD-related pathways	

Even though miRNAs mechanistically differ from small-interfering RNAs (siRNAs) with respect to their multi-targeting potential (Table 2), both classes of small noncoding RNAs are short duplex RNA molecules exerting gene silencing effects at the post-transcriptional level and facing a similar set of barriers for clinical development: delivery issues, poor *in vivo* stability, off-target effects and safety [167].

**Table 2:** Differences between siRNA- and miRNA-based therapeutic agents.

	siRNA	miRNA
**Structure**	Double-stranded RNA duplex, 2 nucleotides 3'-overhang	Double-stranded RNA duplex, 2 nucleotides 3'-overhang
**Target complementarity**	Fully complementary to mRNA target	Partial and imperfect complementarity
**mRNA targets**	One specific target	Multiple context-specific targets
**Mechanism of action**	mRNA cleavage by endonucleolytic capacity	mRNA cleavage
		mRNA decay
		Translational repression
**Clinical application**	Therapeutic agent to knockdown specific mRNA target	Drug target (miRNA mimics & inhibitors)
		Therapeutic agent for the regulation of multiple mRNA targets (miRNA inhibitors)
		Diagnostic tool (biomarkers)

miRNA-based therapeutics mainly comprise synthetic miRNAs used to restore endogenous miRNA levels (e.g. miRNA mimics) or antisense inhibitor oligonucleotides aimed at reducing the functionally available endogenous miRNA (e.g. antamiRs or antagomiRs). While several miRNA-based therapeutics have undergone preclinical testing and have now entered clinical trials for the treatment of a wide variety of pathologies, ranging from cardiac failure to several types of cancer [80, 81, 168–175], none of them is yet targeted towards AD.

Of note, the multi-targeting mode of miRNA action asks for extensive preclinical assessment. A single miRNA can bind several transcripts [23, 176, 177], while one mRNA can be targeted by multiple miRNAs simultaneously. In addition, a miRNA can target mRNAs that exert opposing effects within the same molecular pathway [178]. The relative endogenous abundance of a given miRNA, of other ‘competing’ miRNAs and of their common targets in a particular cell also impacts the strength of the effect of a particular miRNA on its cognate mRNA [21, 179, 180]. Yet, this complex regulatory repertoire may not merely involve ‘random’ transcripts: miRNA targets are often identified within the same molecular pathway(s) and, hence, need to be regulated in a highly coordinated manner [61, 181–185]. Reliable miRNA target identification is a laborious task, further complicated by the high false positive rates of most of the target prediction algorithms [186, 187]. While each miRNA target validation technology bares its own strengths and limitations (systematically reviewed in [188]), the optimization of genome editing tools, such as CRISPR/Cas9 systems, to edit miRNA binding sites *in vivo* holds great promise for direct and more precise functional target validation in the future [189, 190]. Systematic mapping of the genome-wide and cell (type)-specific miRNA targetomes and the affected biological cascades is an absolute requirement for clinical application, and, hence, proteomics and single-cell transcriptomics *in vivo* or high-throughput targetome profiling in human cell lines *in vitro*, are key prior to launching miRNA-based approaches in the clinical development pipeline [191, 192]. Recent studies performing extensive preclinical evaluation of miRNAs, have deployed a so-called ‘miRNA pharmacodynamic signature’ to assess dose-dependent target engagement *in vivo* [80, 81]. Using a panel of previously identified, robustly affected miRNA targets was shown to be an effective measure of miRNA activity in animal models, which considers the functional significance of network regulation by miRNAs. For human-validated targets, such approaches could also be considered in the clinic, if the target panel can be reliably measured in clinically relevant biological fluids, such as blood or CSF. Nevertheless, while this strategy would assess engagement of relevant targets, it would still not address genome-wide disease-relevant and non-disease-relevant effects.

In a recent small-cohort, phase I clinical trial with CDR132L, an anti-miR-132 oligonucleotide for the treatment of heart failure, besides the primary outcomes of safety, target engagement was also considered as secondary outcome [193]. Even though due to ethical constraints this information was derived from prior preclinical studies in large animals [80, 194], it still enabled the prediction of the effective human dose based on plasma miR-132 levels after administration of CDR132L [193].

On- and off-target side effects are currently a major hurdle for miRNA-therapeutics to overcome in order to transit to the clinic. The usage of miRNA therapeutics in combination with other drugs or miRNAs that target the same gene networks can reduce dose and eliminate on-target toxicity [195, 196]. Conversely, off-target toxicity can occur through activation of the immune system by administered double-stranded RNA molecules and the often highly positively charged delivery vehicles [197, 198]. In 2016, a phase I clinical trial testing a liposomal miR-34a mimic (MRX34) for the treatment of advanced solid tumors was halted due to the adverse immune reactions reported in a subset of patients, even though anti-tumor effects were observed [199]. While it is unclear whether the toxicity was caused by the vehicle or the oligonucleotide *per se*, these results underscore the importance of careful dosage titration and preclinical safety assessment with well-designed, standardized toxicity studies in multiple preclinical species.

Efficient and targeted delivery and cellular uptake of RNA therapeutics will be key in eliminating unwanted side effects, as it can significantly limit systemic exposure and high dose requirements. Of note, the intrinsic properties of miRNAs, including their hydrophilic nature, negative charge and relatively high molecular weight, render them poorly permeable across biological membranes [167]. Recent technological advances and insights from the cancer field have paved the way for the development of several distinct DNA and RNA delivery vehicles for transport to the brain, either by crossing the blood-brain-barrier (BBB) or by direct delivery into the CNS. Viral approaches, and in particular recombinant AAV-based systems are a potent CNS delivery platform, which can target specific tissues or cell types (making use of different capsids and promotors) and are safe in the clinic demonstrating durable transgene expression [200]. Intrathecal delivery of AAVs containing an artificial miRNA suppressing superoxide dismutase 1 (SOD1) (a genetic cause of familial amyotrophic lateral sclerosis (ALS)), was shown to be efficient in SOD1 gene silencing in nonhuman primates, yet it did induce peripheral immune responses [201]. Recently, a single-dose gene replacement therapy (Zolgensma) for spinal muscular atrophy (SMA) based on an AAV9 delivery system that can cross the BBB, was approved by the FDA [202]. However, long-term safety studies and improvement of the efficacy of AAV-mediated gene delivery are required, as high doses (10^14^ viral genomes/kg) of AAV9 are necessary to transduce around 20% of the target cell population in the brain, leading to numerous adverse side-effects in animal models and in humans, such as neutralizing antibodies, elevated serum aminotransferase levels and liver toxicity [202–207].

Besides viral gene delivery, non-viral strategies are also emerging for brain targeting of small RNA molecules. Most of the existing knowledge here stems again from siRNA applications, although some preliminary evidence on miRNAs has been reported. miRNA encapsulation into nanoparticles could offer novel opportunities for controlled and putatively cell type-specific miRNA delivery into the brain for therapeutic purposes, either by systemic or direct CNS administration [208, 209]. Advantages of nanoparticle-mediated delivery include increased molecular stability of the RNA payload, protection against endogenous nucleases and the possibility for cell type-specific targeting. Lipid nanoparticles are the most intensively investigated and were the ones approved by the FDA in 2018 for siRNA delivery to the liver [210]. We recently reported the feasibility of nanoparticle-encapsulated miR-132 mimic delivery via intranasal administration into the brain of an AD mouse model [211]. However, assessment of the expression levels of a limited set of predicted targets did not yield consistent results. Systematic functional validation to address broad target engagement *in vivo* is still pending. Key remaining challenges include the widely reported nanoparticle immunogenicity, nonspecific uptake, rapid clearance by macrophages and peripheral toxicity as a result of inefficient targeted delivery [208, 212].

The use of administration routes, such as intracerebroventricular, intrathecal or intranasal infusion, for direct delivery into the CNS, can enable the functional delivery of ‘naked’ oligonucleotides (in the absence of any delivery vehicle) both in mice and humans [134, 213–216]. Chemical modification of the RNA backbone is often required in this case, to augment stability and half-life. A very recent report by the group of Don Cleveland, demonstrated a high efficiency versus toxicity ratio for a novel modified, naked antisense oligonucleotide (ASO) intracerebroventricularly administered in mice, targeting polypyrimidine tract binding protein 1 and inducing the generation of new neurons in the aged mouse dentate gyrus [217]. Evidently, targeted delivery of oligonucleotides is key in order to improve efficacy and safety. For CNS applications, antibody- or peptide- oligonucleotide conjugates may offer a promising strategy for targeted delivery of RNA-based therapeutics across BBB and to specific brain cell types [218–222]. While similar strategies are chemically applicable to a wide range of oligonucleotides, this knowledge emerges once again from nucleotides other than miRNAs, like siRNAs.

Poor stability once inside the cellular environment is another major obstacle towards successful clinical development [191, 192, 223]. Naked, unmodified RNAs are degraded rapidly after administration by the abundant cellular and serum nucleases, resulting in a short half-life *in vivo* [167]. RNA molecules are additionally highly reactive, and hence unstable, due to the presence of the 2’—OH chemical group in the ribose sugar [136]. Chemically modified miRNA mimics or inhibitor oligonucleotides are routinely manufactured commercially and employed to improve stability and binding affinity. In particular, the addition of phosphorothioate nucleotides and methyl groups to the RNA backbone shows a significant increase in protection against nucleases and binding affinity *in vivo* [192, 223, 224]. Moreover, introducing locked-nucleic acid (LNA) modified nucleotides into anti-miRNA oligonucleotides greatly improves their miRNA-targeting efficacy and has been proven safe in non-human primates this far [225–228].

The knowledge gained over the last ten years on miRNA biology and synthetic oligonucleotide technologies only emphasizes the need for systematic preclinical efficacy and safety assessment of miRNA-based AD therapeutics in disease-relevant models. While miRNA-based strategies may offer certain advantages over other therapeutic approaches in AD (Table 1), the existing limitations of this type of therapeutics remain to be critically addressed.

## RNA-based therapeutics in the clinic

RNA-based therapeutics are emerging as a potent new class of drugs in various clinical fields, including neurodegeneration. While extremely promising, these efforts do not yet include miRNA-based strategies targeted against AD pathology.

The first siRNA drug (Patisiran) was approved by the FDA in 2018 for the treatment of hereditary transthyretin-mediated amyloidosis. The siRNA is encapsulated in lipid nanoparticles directing it to the liver after systemic administration, where it binds the mRNA of transthyretin and prevents the production of the mutant protein [229–231]. Groundbreaking advances recently led to the first FDA-approved drug for SMA, a rare neuromuscular disorder. Treatment for SMA was approved at the end of 2016 using a 2′-O-2-methoxyethyl phosphorothioate-modified ASO that is administered through intrathecal infusion. The ASO interferes with the splicing of SMN2 mRNA, thereby increasing the amount of functional SMN2 protein, which can compensate for the loss of SMN1 [213, 214]. More recently, another RNA-based therapy (Zolgensma) was approved by the FDA for SMA, wherein a functional copy of SMN1 gene is delivered using an AAV9 delivery system, enabling durable SMN1 expression following a single intravenous injection [202]. In AD, the most advanced RNA-based therapeutic is based on translational inhibition of TAU mRNA also using an ASO-based approach (NCT03186989, BIIB080, IONIS-MAPT_RX_), and is currently at clinical trial phase I/II. Most notably, over the last year the world has witnessed the first mRNA vaccines to enter the clinic, developed my Moderna and Pfizer against the SARS-CoV-2 virus [232, 233].

The miRNA-targeted pharmaceutical market is less advanced with numerous clinical trials currently underway (CDR132L, Cardior Pharmaceuticals GmbH; RG-012, Genzyme/Sanofi/Regulus Therapeutics; MRG-106, MRG-110, MRG-201, miRagen/Viridian Therapeutics; TargomiRs [172, 234–236]), yet none of them in AD. Miravirsen, an LNA-modified inhibitor of miR-122 with modified phosphorothioate backbone, to treat hepatitis C infection in the liver, was the first anti-miRNA drug to enter the clinic [225, 228]. Miravirsen naturally accumulates in the liver (as a modified small RNA molecule) and therefore does not require a special delivery strategy. This facilitates its application in hepatitis patients but presents a major issue for brain delivery of similar formulations. Following successful initial clinical trials [237, 238], additional phase II clinical trials were performed by Roche. Even though the viral load did decrease in patients, further clinical development was ended due to undisclosed reasons [239]. Similarly, the clinical development of another anti-miR-122 therapy (RG-101) for the treatment of hepatitis C infection, developed by Regulus Therapeutics, was halted in 2017 due to high levels of bilirubin found in the blood of some participants of the phase II clinical trial [240]. Yet, steps towards the right direction are made also for brain diseases. Regulus Therapeutics announced in 2019 the successful termination of preclinical development of RGLS5579, an ASO to inhibit miR-10b, in the treatment of a highly aggressive brain cancer, glioblastoma multiforme. In combination with temozolomide, the inhibition of miR-10b had a synergistic effect and increased the median survival of a glioblastoma chimeric mouse model from 27% to 159% [241]. Similarly, MRG-107, a miR-155 inhibitor has been preclinically validated by miRagen Therapeutics against ALS [242]. Preparations for a phase I clinical trial are ongoing for both RGLS5579 and MRG-107, however, no information on the delivery strategy has been disclosed to date. This far, the preliminary outcomes of the clinical testing of miRNA therapeutics largely suggest that the delivered oligonucleotides can reach their target sites and can also exert functional effects. However, the suspension or discontinuation of some of these clinical trials (e.g. MRX34, Miravirsen, RG-101), calls attention to putative miRNA-specific risks, which may, at least partially, be explained by issues related to multi-targeting-related toxicity. Once again, these observations emphasize the unquestionable necessity for systematic preclinical targetome profiling and for deep understanding of the mechanistic action of miRNA-based drugs, akin to the process followed in the case of any other modality with polypharmacological potential. Interestingly, multitargeted therapeutics modulating gene expression, such as drugs targeting nuclear receptors/transcription factors or epigenetic enzymes, are already used in the clinic and can provide valuable knowledge in that regard [243, 244]. Notably, while the drop-out rate of miRNA therapeutics in clinical trials does not dramatically differ from that of siRNA-based drugs (50% versus 35,38%), there is a significant difference in the number of miRNA and siRNA formulations that enter the clinical pipeline, with over six times more siRNA target drugs [245]. Even though it is not clear whether this is attributed to negative or inadequate evaluation, there is an evident need for intensification of preclinical research on miRNAs.

Although several clinical trials are ongoing to test miRNA-based therapeutics against several peripheral diseases, no such formulations have reached clinical trials so far for the treatment of AD. Nevertheless, gemfibrozil, a previously FDA-approved drug to decrease cholesterol and lipids, has undergone a phase I trial to assess its ability to increase miR-107 levels for prevention of AD in cognitive healthy and MCI individuals (NCT02045056). 48 control and 24 MCI individuals were treated with gemfibrozil or placebo and gemfibrozil appeared safe, inducing a change in miR-107 plasma levels. The secondary outcomes, including CSF Aβ_42_, pTAU, Aβ_42_/pTau ratio, brain atrophy and plasma TNF-α levels, did show trends for a decrease in the treatment group, however, these measures did not reach statistical significance [246]. While potentially promising, such studies do not directly address efficacy and toxicity of miRNA-based oligonucleotides in AD. Future clinical studies of adequate power may lay the groundwork for further miRNA-relevant drug repurposing or development in AD.

## Conclusions and future perspectives

The paradigm shift from the reductionist view ‘one target – one disease’ to the endophenotype network-informed strategy ‘multiple targets – multiple disease pathways’ has started influencing pharmacological approaches against complex multigenic disorders. Network biology and multi-modal therapies begin to attract attention also in AD research [10, 13, 17, 18]. miRNA-targeted therapeutics are particularly suited for such purposes, as they regulate multiple components of several molecular cascades converging on disease-relevant patho-phenotypes. However, as emphasized in this review, the clinical application of miRNAs in brain diseases faces distinct challenges, reflected in the current scarcity of miRNA-based clinical trials in neurodegeneration in general and in AD in particular.

The field of miRNA-based therapeutics is developing in the slipstream of other oligonucleotide-based therapeutics (siRNA, ASO). Further basic research first, to better characterize how miRNAs target pathways of interest, and second, to systematically map on- and off-target toxic effects, is a prerequisite for effective clinical application in AD and other neurodegenerative disorders. Targeted brain delivery and additional investigation of the tolerability of miRNA supplementation or inhibition strategies are key issues to address. The acquisition of miRNA-based companies by major pharmaceutical companies could signal that prime time is approaching for this novel category of drugs [136]. However, despite their promise, as discussed here, application of miRNA therapeutics in AD is lagging behind other disease areas, like cancer, which represents a more generic discrepancy between the two fields, with approximately 30 times more new molecular entities in clinical trials in cancer than in AD [247]. Technology maturation, but also more aggressive investment in the AD field, are needed to bridge the valley between promising initial miRNA research and clinical application [248], very much similar to the path previously followed with other therapeutic approaches, such as monoclonal antibodies. With several companies focusing on miRNA preclinical, clinical and large screening studies, the next years will put these newly emerging approaches to the test and will define how far miRNA biologics are from clinical practice in AD, and whether the many miRNA targets could turn out to be ‘too many’ for clinical application.

## Data Availability

Data sharing is not applicable to this article as no datasets were generated or analyzed during the current study.

## Abbreviations

AD: Alzheimer’s disease
Aβ: β amyloid
pTAU: Phosphorylated TAU protein
miRNA: microRNA
mRNA: Messenger RNA
CNS: Central nervous system
NF-κB: Nuclear factor kappa-B
APP: Amyloid precursor protein
APOE4: Apolipoprotein E4
FTD: Frontotemporal dementia
BACE1: β-secretase
AAV: Adeno-associated virus
CSF: Cerebrospinal fluid
piRNA: Piwi-interacting RNA
MCI: Mild cognitive impairment
FDA: Federal drug agency
siRNA: Small-interfering RNA
BBB: Blood-brain-barrier
SOD1: Superoxide dismutase 1
ALS: Amyotrophic lateral sclerosis
SMA: Spinal muscular atrophy
ASO: Antisense oligonucleotide
LNA: Locked-nucleic acid
